# Non-capsulated *Neisseria* meningitidis sepsis in a paroxysmal nocturnal hemoglobinuria patient treated with ravulizumab: case report and review of the literature

**DOI:** 10.3389/fimmu.2023.1269325

**Published:** 2023-10-02

**Authors:** Nicole Galli, Loredana Pettine, Mauro Panigada, Laura Daprai, Grazia Suriano, Anna Grancini, Wilma Barcellini, Bruno Fattizzo

**Affiliations:** ^1^ Ematologia, Fondazione IRCCS Ca’ Granda Ospedale Maggiore Policlinico, Milan, Italy; ^2^ Department of Oncology and Hemato-Oncology, University of Milan, Milan, Italy; ^3^ Anestesia e Terapia Intensiva Adulti, Fondazione IRCCS Ca’ Granda Ospedale Maggiore Policlinico, Milan, Italy; ^4^ Laboratorio di Microbiologia, Fondazione IRCCS Ca’ Granda Ospedale Maggiore Policlinico, Milan, Italy; ^5^ Laboratorio Analisi, Fondazione IRCCS Ca’ Granda Ospedale Maggiore Policlinico, Milan, Italy

**Keywords:** paroxysmal nocturnal hemoglobinuria, eculizumab, ravulizumab, Neisseria meningitides, proximal complement inhibitors

## Abstract

Paroxysmal nocturnal haemoglobinuria (PNH) is a rare acquired haematopoietic stem cell disease characterized by complement-mediated intravascular hemolysis, thrombosis, and bone marrow failure. Eculizumab and ravulizumab are anti-C5 monoclonal antibodies that reduce hemolysis, anaemia and thrombotic risk, but are associated with increased risk of infection with encapsulated bacteria, including *Neisseria meningitidis.* We report a case of life-threatening infection by non-groupable *Neisseria meningitidis* in a young PNH patient treated with ravulizumab. Despite prompt admission to the intensive care unit, microbe isolation was delayed due to the negativity of capsular antigens, and the patient required intubation, dialysis, and transfusion support for pancytopenia. Notably, PNH disease activity remained controlled and no additional anti-C5 doses were administered. Increasing awareness regarding septic risk in PNH patients on complement inhibitors despite vaccinations is pivotal. A warning about serotypes generally not pathogenetic and not covered by vaccination, such as non-capsulated forms, is emerging.

## Introduction

Paroxysmal nocturnal hemoglobinuria (PNH) is a rare disease characterized by acquired mutation in the PIGA gene in hematopoietic stem cells making red blood cells susceptible to complement mediated hemolysis (1, 2).

Eculizumab and ravulizumab are anti-C5 monoclonal antibodies that inhibit the terminal complement system, reducing hemolysis, anaemia, occurrence of thrombosis and PNH-related mortality. Complement inhibitors have a warning regarding the increased risk of infection with encapsulated bacteria, especially *Neisseria meningitidis*, reaching up to 2000-fold the risk of the general population (3).

The cumulative reporting rate of meningococcal infection in eculizumab-treated patients tended to decline over time following the initial approval of the drug, then remained relatively stable over the most recent years, at approximately 0.25 per 100 patient-years (4). For the newer anti-C5 ravulizumab the shorter follow-up does not allow clearcut epidemiologic evaluation, and only 1 case of *Neisseria* sepsis has been reported (5).

For patients on anti-complement therapy, there are no available guidelines regarding prevention strategies, accounting for a wide heterogeneity across different countries. Expert consensus suggest administering conjugated ACYW135 and serogroup B vaccines, whilst the use of long-term antibiotic prophylaxis is debated (3). Here we report a rare case of life-threatening infection by non-groupable *Neisseria meningitidis* in a young PNH patient treated with ravulizumab along with a review of the literature.

## Methods

The investigation was conducted according to the Helsinki declaration and the patient gave informed consent. All information regarding patient clinical history and hematologic parameters were systematically collected from initial presentation (March 2023) until the time of writing.

## Case description

A 25-years-old man of North-African ancestry was diagnosed in November 2021 with classic hemolytic PNH. Previous medical history included diagnosis of severe aplastic anaemia at the age of three treated with androgens and cyclosporin. After vaccination against *N. meningitidis* with tetravalent conjugate vaccine (MenACWY) in December 2021 and with anti-serogroup B vaccine (4CMenB) in January 2022, the patient was started on ravulizumab in March 2022 obtaining a major response with Hb>10 g/dL and LDH normalization.

**Figure 1:**
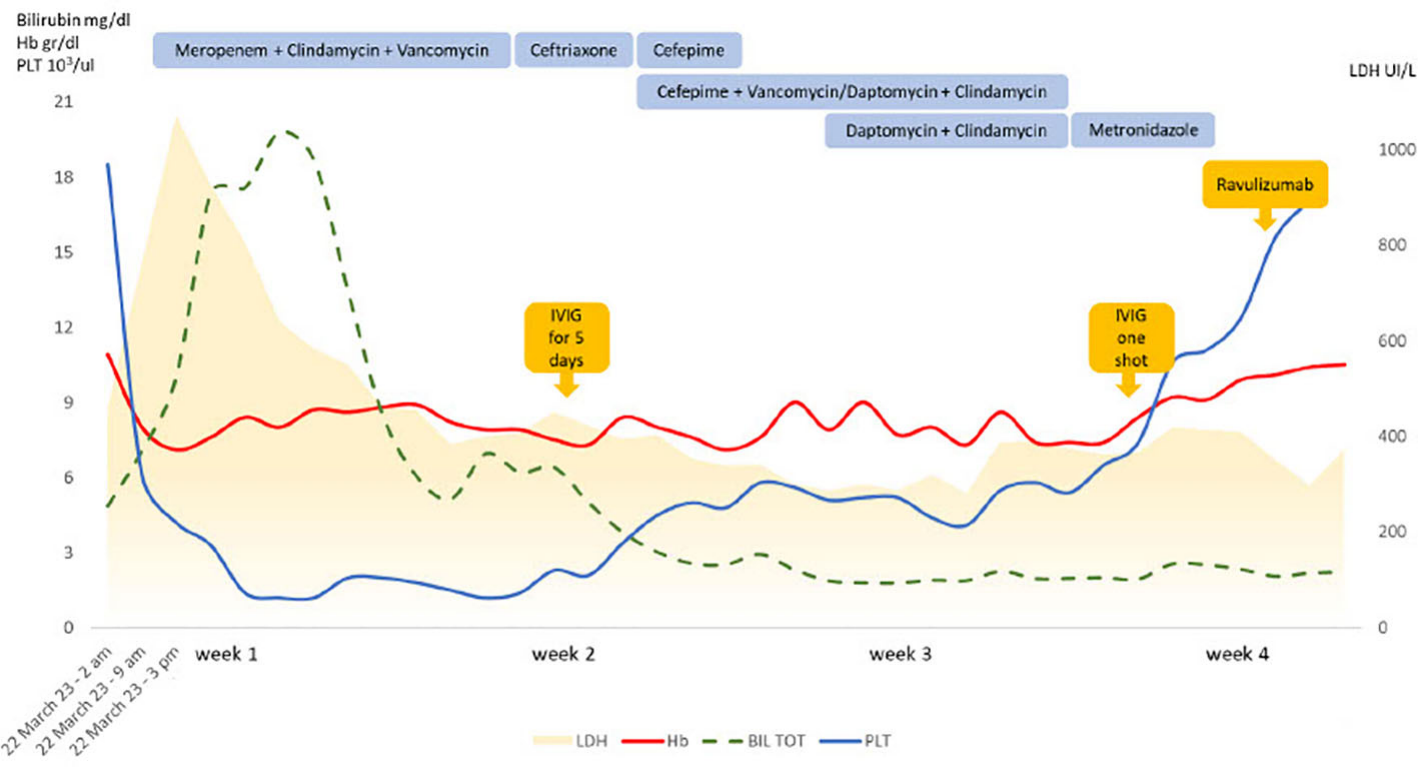
Laboratory features and treatments during the follow up. IVIG, intravenous immunoglobulin; Hb, hemoglobin; PLT, platelets; LDH, lactate dehydrogenase; BIL TOT, total bilirubin.

In March 2023, he was admitted to the emergency department complaining fatigue, malaise, abdominal pain and loose stools since about three days. Clinical examination showed tachycardia (130/min), fever (up to 40°C) and hypotension (70/40 mmHg). Laboratory evaluation revealed lactic acidosis (blood gas analysis showing lactate 13 mmol/l, pH 7.1), a septic constellation (procalcitonin >100 mg/L [normal range: 0.02-0.06], CRP 33.28 mg/dl [< 0.5]) with thrombocytopenia (42 x 10^3^/uL [130– 400]) and disseminated intravascular coagulation (DIC). Additionally, brisk LDH increase was noted, accompanied by mild unconjugated hyperbilirubinemia and progressive decrease of Hb values (**Figure 1**). Last ravulizumab infusion dated 4 weeks back (February 2023). The patient was transferred to the intensive care unit (ICU). Volume resuscitation, vasoactive amins and anti-infective treatment with meropenem, vancomycin and clindamycin were started immediately after sampling blood cultures. Abdominal computed tomography showed ascites and thickening of the intestinal loops, without signs for Waterhouse-Friedrichsen syndrome (surrenal glands necrosis associated with *Neisseria* infections). General conditions rapidly deteriorated with shock status, acute renal failure and acute respiratory distress syndrome (ARDS) and he was intubated and mechanically ventilated. Renal replacement therapy with continuous veno-venous haemodialysis was also started. Thereafter, Hb and LDH remained stable, and no additional anti-complement therapy was administered. Due to markedly reduced neutrophils and platelets along with muco-cutaneous bleeding requiring plasma and platelet transfusions, intravenous immunoglobulins 0,4 g/Kg Day over five consecutive days were administered along with a single injection of granulocyte-colonies stimulating factor (G-CSF). This led to hematologic improvement with neutrophils normalization and platelets increase to >50x10^3^/uL.

**Figure 2:**
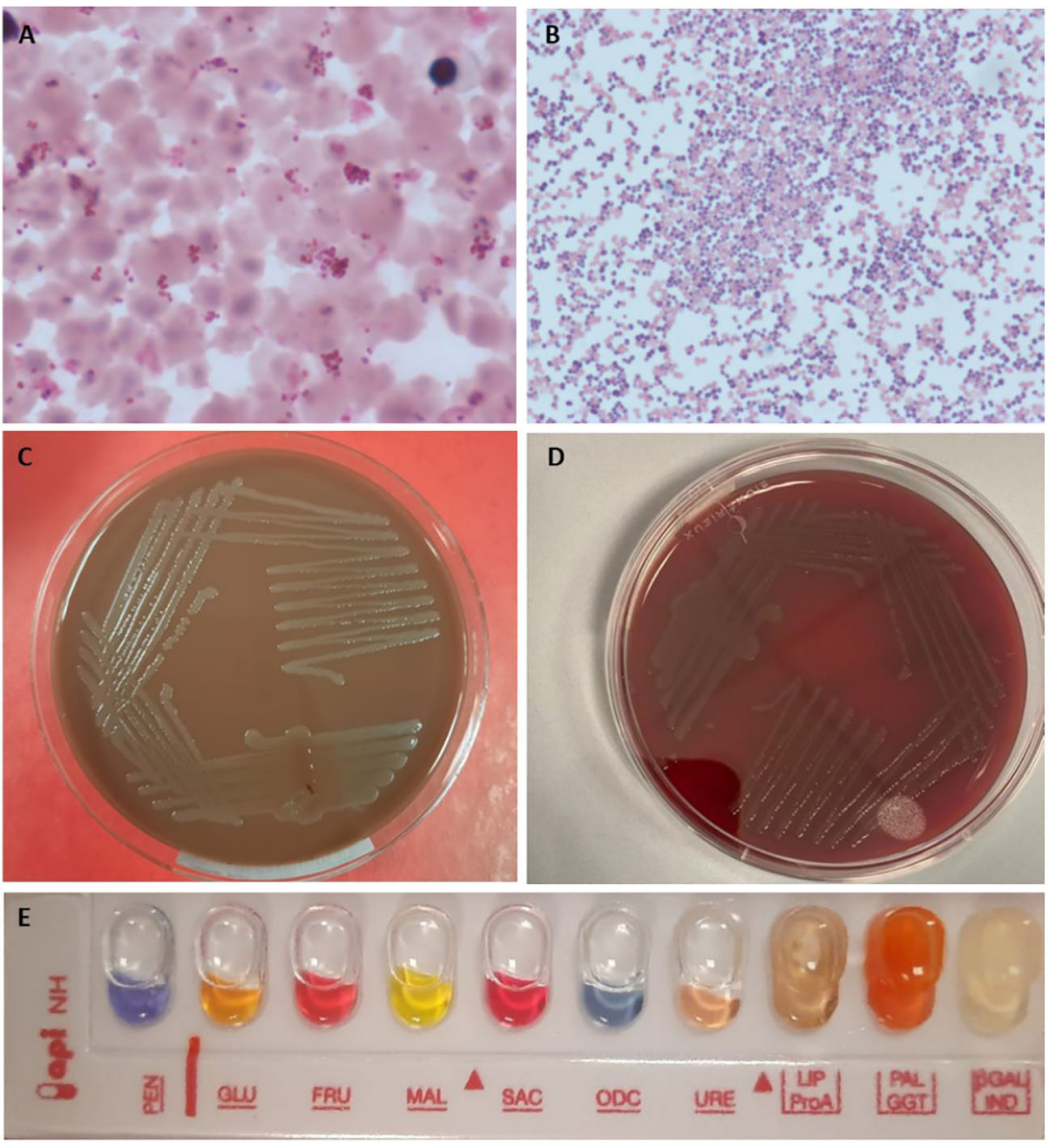
Blood cultures and biochemical tests for Neisseria meningitidis. **(A, B)** blood culture medium broth and colture smear by Gram staining 100x; **(C)** Chocolate-agar culture plate; **(D)** Blood-Agar culture plate; **(E)** biochemical identification gallery api NH.

Blood smear from blood cultures (**Figures 2A, B**) showed several Gram-negative diplococci but molecular biology for *Neisseria meningitidis* capsular antigens were negative. Notably, bacteria grew on chocolate-agar (**Figure 2C**) and blood-agar plates (**Figure 2D**). Identification by MALDI-TOF mass spectrometry Vitek MS was performed and subsequentl biochemical gallery api NH Bio Merieux confirmed the diagnosis of N. meningitidis (**Figure 2E**). The strain was not groupable with specific antisera and PCR method and it was sent to the National Reference Center - Istituto Superiore di Sanità (ISS) for sequencing.

The susceptibility test was performed and the strain showed sensitivity to ceftriaxone, meropenem and rifampicin, thus ceftriaxone and later cefepime were started. Three weeks from initial admission, due to persistent fever with worsening thrombocytopenia, bone marrow aspirate was performed and reduced cellularity consistent with previous aplastic anemia and inflammatory septic state was found, whilst hemophagocytic lymphohistiocytosis was ruled out. Anti-infective treatment was changed, and a second course of intravenous immunoglobulins (19 April 2023) was given with progressive hematologic improvement.

During ICU stay additional complications occurred, including invasive candidiasis, dental granuloma due to *Prevotella denticula*, and delirium.

After 4 weeks patient conditions markedly improved and renal and respiratory functions progressively normalized. PNH-clone was re-evaluated and confirmed to be 97% on granulocytes, and ravulizumab therapy was given at 6 weeks after initial admission (10 weeks from last infusion, allowing for the recovery of the septic state and exploiting the 7-day therapeutic window of ravulizumab). During ICU stay anticoagulation was not performed because of severe thrombocytopenia. Later (28 April 2023) thoracic computed tomography showed subsegmental pulmonary embolism, and anticoagulation with low molecular weight heparin was started. The patient was discharged after 7 weeks of admission (9 May 2023) in good general conditions, normal PLT and neutrophil counts, Hb 9.8 g/dL, and LDH 1.2 x upper limit of normality (ULN).

## Review of the literature

**Table 1** summarizes available reports on *Neisseria* infection in PNH patients treated with complement inhibitors (5–11).

**Table 1:** Literature review of infections occurring during anti-complement therapy for paroxysmal nocturnal hemoglobinuria (PNH).

Reference	N° of patients	Type of PNH	Type of Complement Inhibitor (treatment duration)	Infection agent	Outcome
Hawkins Kl et al, 2017 (6)	1 (F, 18)	Classic PNH	Eculizumab (24 months)	*Neisseria meningitidis, non-groupable*	Resolved
Hernando Real at al, 2017 (7)	1 (M, 23)	Classic PNH	Eculizumab (12 months and 48 months)	*Neisseria meningitidis* (serogroup B)	Resolved
Nolfi-Donegan et al, 2018 (8)	1 (F, 16)	Classic PNH	Eculizumab (28 days)	*Neisseria meningitidis*, non-groupable	Died
Reher et al, 2018 (9)	1 (F, 26)	Classic PNH	Eculizumab (72 months)	*Neisseria meningitidis*	Resolved
Crew et al, 2019 (10)	9 (8 F, 1 M, median age 23, range 18-44)	5 classic PNH, 1 PNH associated with AA, 3 HUS	Eculizumab	*Neisseria gonorrhoeae*	8 resolved, 1 died
Crew et al, 2019 (11)	7 (4 F, 3 M; median age 17, range 4-38)	2 classic PNH, 1 PNH associated with AA, 3 HUS, 1 CAPS	Eculizumab	2 *Neisseria mucosa/subflava*, 2 *Neisseria mucosa*, 2 *Neisseria cinerea*, 1 *Neisseria subflava*	7 resolved
Z. Yu et al, 2020 (5)	1 (M, 24)	Classic PNH	Ravulizumab (36 months)	*Neisseria gonorrhoeae*	Resolved

A total of 21 patients have been described, 15 females and 6 males, with a median age of 23 years, ranging from 4 to 44 years. All patients were receiving eculizumab at the time of infection except one, who had received ravulizumab. The reports included 2 cases of *N. meningitidis*, 2 of *N. meningitidis* non-groupable, 10 of *N. gonorrhoeae*, 2 of *Neisseria mucosa/subflava*, 2 of *Neisseria mucosa*, 2 of *Neisseria cinerea* and 1 of *Neisseria subflava*. Among these, only one case was associated with onset of break through hemolysis (BTH), which was absent in 3 cases and not detailed in the remaining reports.

All patients reported recovered, except for two, who died. Major risk factors emerged were young age and unprotected sexual contact.

More specifically, the first report (6) highlights a life-threatening case of meningococcal disease caused by a non-groupable strain and complicated by purpura fulminans. A similar pathogen was seen in a young girl presenting with Waterhouse-Friedrichsen syndrome after her second dose of eculizumab (8). By whole-genome sequencing, the meningococcal strain isolated was found to be non-groupable with a capsule null locus, which is generally observed in asymptomatic carriers. Whole-genome sequencing demonstrated a capsule null locus, and the Authors showed that *in-vitro* killing of such strain was inhibited by eculizumab (8).

Rare cases of septic shock due to infection with *Neisseria meningitidis* serogroup B despite prior vaccination with 4CMenB have also been described (7, 9). Hernando et al., reported a case of a young man with sepsis due to *N. meningitidis* serogroup B, in which molecular analysis unravelled different expression of surface antigens not included in the vaccine (7). The second case was a female presenting to the emergency department with a severe meningococcal infection, that required fluid resuscitation, broad spectrum antibiotics, intubation, dialysis and transfusion support. The Authors pointed out that bactericidal antibody titers (serum bactericidal assay using human complement, hSBA) could not be performed in eculizumab treated patients, because the assay is based on exogenous human complement, which is inactivated by the drug (9).

Beyond meningococcal, patients receiving eculizumab may be at higher risk for other *Neisseriae* not covered by available vaccines. Crew et al., described 16 cases of *Neisseria* spp infection in patients receiving eculizumab for PNH and other complement driven diseases such as atypical hemolytic uremic syndrome (aHUS) and catastrophic antiphospholipid antibody syndrome (CAPS) (10, 11). These included 9 cases of *N. gonorrhoeae*, 8 classified as disseminated and 2 presenting with septic shock, 1 requiring mechanical ventilation. All gonococcal infections resolved, except for one case (10). The other 7 cases were caused by typically commensal *Neisseria* spp. (*N. mucosa/subflava, N. mucosa, N. cinerea, N. subflava*). In these series, most patients had comorbidities or comedications additional to eculizumab, justifying immunosuppression, that may have contributed to risk of infection (11).

Even ravulizumab was recently associated with severe gonococcal infection in a 24-years-old man with PNH who developed a disseminated infection after unprotected sex (5).

## Discussion and conclusions

We describe a rare case of severe septic shock due to community-acquired meningococcal infection caused by non-groupable *N.meningitidis* in a young man with PNH treated with ravulizumab.

Compared with the general population, the incidence of meningococcal infections are 1000- to 2000-fold higher in patients receiving anti-complement therapy despite the recommended risk mitigation measures (12). The latter (3) include vaccination with MenACWY and 4CMenB at least two weeks prior to receiving the first dose of anti-C5 drug, and re-vaccination with tetravalent conjugate vaccine every five years. This schedule may vary from country to country in the absence of general guidelines. Meningococcal vaccination may be incompletely effective because of impaired opsonophagocytic (OPA) killing as a consequence of inhibiting cleavage of C5 to release C5a, a potent proinflammatory mediator needed for OPA killing of meningococci (13). Additionally, serogroups covered by vaccines can cause severe disease due to the variety of surface expression of antigens, possibly non-susceptible to the vaccine.

On the other hand, patients with complement impairment are at higher risk for infections due to encapsulated bacteria other than *N. meningitidis*, such as *N. gonorrhoeae*, as reported particularly in young subjects. Most of them presented as disseminated infections, which are rare in the general population. Awareness of the risk of invasive gonococcal infection in patients receiving anti-complement therapy is critical for implementing appropriate infection prevention measures and patient education on risk of sexual contagions. In fact, many infections occurred after unprotected sexual contact, so prescribers should be encouraged to obtain personal histories from patients receiving these therapies to guide appropriate screening recommendations.

Importantly, the risk is less known for novel proximal inhibitors (i.e. C3, factor B and factor D inhibitors) that require broaden vaccination prophylaxis with anti-*Haemophilus* and anti-*Pneumococcus* vaccines, and that were not associated with *Neisseria* infections during the clinical trials although the follow up is still limited to draw conclusions.

Finally, the rate of immune response to current vaccinations is not known, particularly with 4CMenB. Future studies are needed to further address this issue. Prophylaxis with penicillin or ciprofloxacin are recommended at least until immunization is completed, whilst lifelong chemoprophylaxis remains controversial (3).

In conclusion, whilst vaccination strategies are recommended in PNH patients on anti-complement therapies, many drawbacks preclude full infections prevention. The latter include impaired bacterial killing due to complement inactivation, incomplete coverage of bacterial strains, and uncertainty regarding seroconversion after vaccines. Patient education and physician awareness, including internal medicine specialists and general practitioners, remain pivotal to ensure prompt recognition and management of severe infectious complications with current as well as with proximal complement inhibitors. To better profile the risk, continuous monitoring on epidemiology of meningococcal infections is needed, including collection of regional/national registry data.

## Data availability statement

The raw data supporting the conclusions of this article will be made available by the authors, without undue reservation.

## Ethics statement

Written informed consent was obtained from the individual(s) for the publication of any potentially identifiable images or data included in this article.

## Author contributions

NG: Data curation, Formal Analysis, Investigation, Writing – original draft. LP: Writing – review & editing. MP: Writing – review & editing. LD: Writing – review & editing. GS: Writing – review & editing. AG: Writing – review & editing. WB: Formal Analysis, Supervision, Validation, Writing – review & editing. BF: Data curation, Formal Analysis, Project administration, Supervision, Validation, Writing – review & editing.
